# Supplementary material for: Enhancing the Outcome of Crystallographic Fragment Screening by Choosing the Optimal Protein Crystal Form
Source: Small Sci. 2026 Mar 4;6(3):e202500484. doi: 10.1002/smsc.202500484 (PMC12970188; doi:10.1002/smsc.202500484)
Supplement: Supplementary file 1 — Supplementary Material [file SMSC-6-e202500484-s001.pdf]

Supporting Information

Enhancing the Outcome of Crystallographic Fragment Screening by Choosing the Optimal Protein Crystal Form

Tatjana Barthel[1+,], Jan Wollenhaupt[1,2+], Laila S. Benz[1], Patrick Reinke[3], Linlin Zhang[4], Melanie Oelker[1,5], Frank Lennartz[1], Helena Tabermann[3], Uwe Mueller[1], Alke Meents[3], Rolf Hilgenfeld[4] & Manfred S. Weiss[1,*]*

+ shared first authors

* corresponding author: tatjana.barthel@helmholtz-berlin.de, msweiss@helmholtz-berlin.de

1) Macromolecular Crystallography, Helmholtz-Zentrum Berlin, Albert-Einstein-Str. 15, 12489 Berlin, Germany

2) present address: Proteros biostructures GmbH, Bunsenstr. 7a, 82152 Planegg-Martinsried, Germany

3) Deutsches Elektronen-Synchrotron DESY, Biomedical Research with X-rays (FS-BMX), Building 94, Room O4.003, Notkestraße 85, 22607 Hamburg, Germany

4) Institute of Molecular Medicine, University of Lübeck, Ratzeburger Allee 160, Lübeck 23562, Germany

5) Institute of Biology, Humboldt-Universität zu Berlin, Unter den Linden 6, Berlin 10099, Germany

Table S1: Crystallographic data for all fragment-bound structures after PanDDA quick refinement.

	MPro_F2XEntry_sg5_B07 (7HUC)	MPro_F2XEntry_sg5_B08 (7HUD)	MPro_F2XEntry_sg5_D08 (7HUE)
**Wavelength**	0.9184	0.9184	0.9184
**Resolution range**	21.84 - 1.52 (1.54 - 1.52)	21.78 - 1.61 (1.63 - 1.61)	20.64 - 1.7 (1.72 - 1.7)
**Space group**	C 1 2 1	C 1 2 1	C 1 2 1
**Unit cell**	113.4 53.09 44.41 90 102.58 90	112.55 52.81 44.69 90 102.65 90	112.35 53.06 44.81 90 102.21 90
**Total reflections**	224,178 (37,156)	188,237 (30,990)	159,285 (26,129)
**Unique reflections**	39,298 (1,286)	33,046 (1,045)	28,400 (921)
**Multiplicity**	5.7 (5.9)	5.7 (5.9)	5.6 (5.7)
**Completeness (%)**	99.0 (97.7)	99.4 (98.5)	99.7 (98.9)
**Mean I/sigma(I)**	12.9 (0.9)	12.2 (0.9)	13.6 (1.1)
**Wilson B-factor**	24.5	28.1	30.1
**R-merge**	0.081 (1.773)	0.087 (1.768)	0.071 (1.53)
**R-meas**	0.089 (1.944)	0.096 (1.941)	0.079 (1.684)
**CC1/2**	0.999 (0.486)	0.999 (0.363)	0.999 (0.481)
**Reflections used in refinement**	39,298 (1,286)	33,046 (1,045)	28,400 (921)
**Reflections used for R-free**	1,972 (142)	1,658 (121)	1,424 (104)
**R-work**	0.203	0.199	0.198
**R-free**	0.235	0.229	0.233
**Number of non-hydrogen atoms**	2,932	2,500	2,515
**macromolecules**	2,784	2,399	2,417
**ligands**	38	22	26
**solvent**	110	79	72
**Protein residues**	306	306	306
**RMS(bonds)**	0.009	0.158	0.115
**RMS(angles)**	1.6	4.0	3.8
**Ramachandran favored (%)**	98.0	97.0	97.4
**Ramachandran allowed (%)**	1.6	2.6	2.3
**Ramachandran outliers (%)**	0.3	0.3	0.3
**Rotamer outliers (%)**	2.6	3.0	1.9
**Clashscore**	4.49	3.4	3.7
**Average B-factor**	27.1	30.3	33.7
**macromolecules**	27.1	30.2	33.6
**ligands**	26.3	38.1	39.5
**solvent**	28.3	31.0	35.1

	MPro_F2XEntry_ sg19_B05 (7I13)	MPro_F2XEntry_ sg19_B08 (7I14)	MPro_F2XEntry_ sg19_C02 (7I15)	MPro_F2XEntry_ sg19_C07 (7I16)
**Wavelength**	0.9184	0.9184	0.9184	0.9184
**Resolution range**	45.97 - 2.0 (2.05 - 2.0)	46 - 1.95 (2 - 1.95)	49.38 - 2.0 (2.05 - 2.0)	45.74 - 2.13 (2.19 - 2.13)
**Space group**	P 21 21 21	P 21 21 21	P 21 21 21	P 21 21 21
**Unit cell**	68.19 99.76 103.6 90 90 90	67.99 99.91 103.65 90 90 90	67.98 99.62 103.7 90 90 90	67.67 98.94 103.18 90 90 90
**Total reflections**	285,199 (47,097)	297,835 (49,002)	275,616 (46,960)	222,723 (36,588)
**Unique reflections**	48,132 (3,162)	51,764 (3,422)	48,179 (3,137)	38,744 (2,730)
**Multiplicity**	5.9 (6.1)	5.8 (5.9)	5.7 (6.1)	5.8 (6.0)
**Completeness (%)**	99.6 (99.8)	99.8 (99.5)	99.8 (99.7)	98.4 (98.3)
**Mean I/sigma(I)**	8.9 (1.3)	6.8 (1.1)	6.4 (1.1)	6.8 (1.2)
**Wilson B-factor**	30.2	31.0	28.7	34.4
**R-merge**	0.154 (1.33)	0.143 (1.167)	0.225 (1.444)	0.201 (1.464)
**R-meas**	0.168 (1.455)	0.184 (1.525)	0.247 (1.575)	0.218 (1.583)
**CC1/2**	0.996 (0.496)	0.994 (0.463)	0.992 (0.408)	0.992 (0.501)
**Reflections used in refinement**	48,132 (3,162)	51,764 (3,422)	48,179 (3,137)	38,744 (2,730)
**Reflections used for R-free**	2,100 (138)	2,100 (139)	2,099 (136)	1,938 (136)
**R-work**	0.228	0.230	0.241	0.227
**R-free**	0.268	0.270	0.279	0.270
**Number of non-hydrogen atoms**	5,116	5,490	5,726	4,867
**macromolecules**	5,008	5,357	5,603	4,802
**ligands**	26	64	63	27
**solvent**	82	69	60	38
**Protein residues**	606	601	602	607
**RMS(bonds)**	0.014	0.014	0.014	0.013
**RMS(angles)**	1.7	1.7	1.7	1.7
**Ramachandran favored (%)**	95.9	96.8	96.7	95.5
**Ramachandran allowed (%)**	3.8	3.0	3.2	4.3
**Ramachandran outliers (%)**	0.3	0.2	0.2	0.2
**Rotamer outliers (%)**	2.3	1.8	4.3	2.2
**Clashscore**	2.6	5.9	4.4	4.6
**Average B-factor**	36.8	36.0	34.5	40.3
**macromolecules**	36.9	36.2	34.5	40.4
**ligands**	27.8	32.2	42.6	37.4
**solvent**	31.6	31.3	27.1	32.8

	MPro_F2XEntry_sg19_C10 (7I17)	MPro_F2XEntry_sg19_D04 (7I18)	MPro_F2XEntry_sg19_D08 (7I19)	MPro_F2XEntry_sg19_D11 (7I1A)
**Wavelength**	0.9184	0.9184	0.9184	0.9184
**Resolution range**	46 - 1.69 (1.73 - 1.69)	45.89 - 2.0 (2.04 - 2.0)	46.13 - 1.93 (1.97 - 1.93)	49.2 - 1.73 (1.77 - 1.73)
**Space group**	P 21 21 21	P 21 21 21	P 21 21 21	P 21 21 21
**Unit cell**	67.89 99.84 103.65 90 90 90	68.05 99.28 103.5 90 90 90	68.06 99.89 104.03 90 90 90	67.83 99.09 103.14 90 90 90
**Total reflections**	453,419 (75,927)	277,043 (43,682)	330,015 (51,853)	430,503 (70,047)
**Unique reflections**	78,658 (5,202)	48,237 (3,121)	53,905 (3,555)	71,352 (4,667)
**Multiplicity**	5.8 (6.0)	5.7 (5.7)	5.7 (5.6)	6.0 (6.2)
**Completeness (%)**	99.0 (99.3)	99.9 (99.9)	99.7 (99.8)	97.5 (97.4)
**Mean I/sigma(I)**	8.5 (0.9)	9.0 (1.1)	8.9 (1.2)	12.2 (1.2)
**Wilson B-factor**	24.8	31.4	29.9	24.0
**R-merge**	0.125 (1.647)	0.154 (1.48)	0.143 (1.167)	0.104 (1.521)
**R-meas**	0.137 (1.805)	0.170 (1.631)	0.158 (1.283)	0.114 (1.66)
**CC1/2**	0.997 (0.333)	0.997 (0.456)	0.997 (0.495)	0.999 (0.447)
**Reflections used in refinement**	78,658 (5,202)	48,237 (3,121)	53,905 (3,555)	71,352 (4,667)
**Reflections used for R-free**	2,099 (139)	2,099 (136)	2,093 (138)	2,101 (138)
**R-work**	0.232	0.214	0.2307	0.212
**R-free**	0.253	0.254	0.2684	0.239
**Number of non-hydrogen atoms**	5,275	5,424	5,642	6,115
**macromolecules**	5,111	5,281	5,492	5,881
**ligands**	34	38	62	66
**solvent**	130	105	88	168
**Protein residues**	604	606	602	611
**RMS(bonds)**	0.015	0.014	0.013	0.014
**RMS(angles)**	1.8	1.7	1.6	1.6
**Ramachandran favored (%)**	97.2	97.0	97.7	97.7
**Ramachandran allowed (%)**	2.5	2.7	2.3	2.3
**Ramachandran outliers (%)**	0.3	0.3	0	0
**Rotamer outliers (%)**	3.0	2.5	3.4	2.7
**Clashscore**	4.2	3.6	3.7	5.4
**Average B-factor**	31.6	38.1	36.7	29.5
**macromolecules**	31.7	38.2	36.8	29.6
**ligands**	36.6	37.1	33.6	28.0
**solvent**	29.6	34.0	30.9	29.6

	MPro_F2XEntry_sg19_E11 (7I1C)	MPro_F2XEntry_sg19_F04 (7I1D)	MPro_F2XEntry_sg19_G03 (7I1E)	MPro_F2XEntry_sg19_G04 (7I1F)
**Wavelength**	0.9184	0.9184	0.9184	0.9184
**Resolution range**	40.19 - 1.95 (2 - 1.95)	46.06 - 2.0 (2.05 - 2.0)	45.98 - 1.88 (1.92 - 1.88)	45.78 - 1.77 (1.81 - 1.77)
**Space group**	P 21 21 21	P 21 21 21	P 21 21 21	P 21 21 21
**Unit cell**	68.11 99.56 103.94 90 90 90	67.96 99.47 103.93 90 90 90	67.92 99.59 103.67 90 90 90	67.69 99.33 103.18 90 90 90
**Total reflections**	307,300 (49,769)	286,518 (47,203)	333,013 (51,567)	399,897 (64,014)
**Unique reflections**	51,838 (3,403)	48,092 (3,137)	57,495 (3,746)	68,396 (4,489)
**Multiplicity**	5.9 (6.0)	6.0 (6.2)	5.8 (5.6)	5.9 (5.9)
**Completeness (%)**	99.6 (99.2)	99.5 (99.9)	99.3 (98.7)	99.7 (99.6)
**Mean I/sigma(I)**	10.1 (1.2)	9.3 (1.1)	8.6 (1.1)	10.5 (1.0)
**Wilson B-factor**	31.6	30.2	28.4	26.1
**R-merge**	0.126 (1.364)	0.168 (1.61)	0.142 (1.33)	0.118 (1.668)
**R-meas**	0.138 (1.492)	0.184 (1.759)	0.157 (1.466)	0.130 (1.831)
**CC1/2**	0.997 (0.463)	0.996 (0.380)	0.997 (0.462)	0.998 (0.420)
**Reflections used in refinement**	51,838 (3,403)	48,092 (3,137)	57,495 (3,746)	68,396 (4,489)
**Reflections used for R-free**	2,100 (138)	2,100 (137)	2,099 (137)	2,100 (137)
**R-work**	0.225	0.227	0.219	0.220
**R-free**	0.251	0.268	0.248	0.259
**Number of non-hydrogen atoms**	5,080	5,962	5,030	5,432
**macromolecules**	4,966	5,857	4,903	5,249
**ligands**	28	38	27	38
**solvent**	86	67	100	145
**Protein residues**	602	601	605	602
**RMS(bonds)**	0.014	0.014	0.014	0.01
**RMS(angles)**	1.7	1.7	1.7	1.6
**Ramachandran favored (%)**	97.8	96.1	96.5	96.8
**Ramachandran allowed (%)**	2.2	3.4	3.3	3.0
**Ramachandran outliers (%)**	0	0.5	0.2	0.2
**Rotamer outliers (%)**	2.2	3.5	2.6	2.2
**Clashscore**	4.2	8.8	2.7	3.9
**Average B-factor**	38.1	36.2	35.9	33.5
**macromolecules**	38.2	36.2	36	33.5
**ligands**	29.5	49.9	35.9	44.2
**solvent**	32.5	28.2	30.5	31.2

	MPro_F2XEntry_sg19_G09 (7I1G)	MPro_F2XEntry_sg19_G10 (7I1H)	MPro_F2XEntry_sg19_H03 (7I1I)	MPro_F2XEntry_sg19_H11 (7I1J)
**Wavelength**	0.9184	0.9184	0.9184	0.9184
**Resolution range**	49.28 - 1.83 (1.87 - 1.83)	46.68 - 1.98 (2.03 - 1.98)	45.51 - 1.88 (1.92 - 1.88)	49.47 - 1.85 (1.9 - 1.85)
**Space group**	P 21 21 21	P 21 21 21	P 21 21 21	P 21 21 21
**Unit cell**	67.75 98.55 102.84 90 90 90	68.81 100.7 105.35 90 90 90	67.83 98.84 102.55 90 90 90	68.28 98.8 104.47 90 90 90
**Total reflections**	359,421 (55,530)	293,691 (45,143)	303,524 (40,576)	352,058 (53,678)
**Unique reflections**	61,199 (4,045)	51,335 (3,378)	55,928 (3,606)	59,793 (3,949)
**Multiplicity**	5.9 (5.7)	5.7 (5.5)	5.4 (4.7)	5.9 (5.7)
**Completeness (%)**	99.6 (100.0)	99.8 (99.6)	98.2 (96.7)	98.8 (98.6)
**Mean I/sigma(I)**	10.5 (1.2)	8.7 (1.2)	9.8 (1.0)	7.8 (1.0)
**Wilson B-factor**	28.3	30.9	30.5	27.8
**R-merge**	0.111 (1.426)	0.143 (1.316)	0.116 (1.448)	0.151 (1.599)
**R-meas**	0.122 (1.570)	0.157 (1.454)	0.128 (1.626)	0.165 (1.760)
**CC1/2**	0.998 (0.460)	0.996 (0.468)	0.998 (0.389)	0.996 (0.400)
**Reflections used in refinement**	61,199 (4,045)	51,335 (3,378)	55,928 (3,606)	59,793 (3,949)
**Reflections used for R-free**	2,099 (138)	2,099 (138)	2,099 (135)	2,101 (139)
**R-work**	0.209	0.224	0.219	0.210
**R-free**	0.235	0.258	0.255	0.248
**Number of non-hydrogen atoms**	5,366	4,877	4,873	4,983
**macromolecules**	5,197	4,757	4,768	4,807
**ligands**	51	36	39	39
**solvent**	118	84	66	137
**Protein residues**	605	602	606	611
**RMS(bonds)**	0.014	0.01	0.014	0.009
**RMS(angles)**	1.7	1.7	1.7	1.5
**Ramachandran favored (%)**	97.3	97.3	97.5	97.9
**Ramachandran allowed (%)**	2.5	2.5	2.5	2.1
**Ramachandran outliers (%)**	0.2	0.2	0	0
**Rotamer outliers (%)**	2.4	1.3	1.7	1.1
**Clashscore**	4.5	1.6	3.0	3.0
**Average B-factor**	34.5	36.6	39.2	34.6
**macromolecules**	34.5	36.6	39.2	34.6
**ligands**	33.4	42.2	44.0	42.1
**solvent**	34.3	31.5	33.5	32.9

Table S2: Comparison between orthorhombic MPro crystals between Noske *et al.* (2021) and this study.

	Noske *et al.*	This study orthorhombic
**Protein construct**	Immature MPro (50 amino acids per row)<br>**GAM**SGFRKMAFPSGKVEGCMVQVTCGTTTLNGLWLDDVVYCPRHVICTSE<br>DMLNPNYEDLLIRKSNHNFLVQAGNVQLRVIGHSMQNCVLKLKVDTANPK<br>TPKYKFVRIQPGQTFSVLACYNGSPSGVYQCAMRPNFTIKGSFLNGSCGS<br>VGFNIDYDCVSFCYMHHMELPTGVHAGTDLEGNFYGPFVDRQTAQAAGTD<br>TTITVNVLAWLYAAVINGDRWFLNRFTTTLNDFNLVAMKYNYEPLTQDHV<br>DILGPLSAQTGIAVLDMCASLKELLQNGMNGRTILGSALLEDEFTPFDVV<br>RQCSGVTFQ	Mature MPro (50 amino acids per row)<br>---SGFRKMAFPSGKVEGCMVQVTCGTTTLNGLWLDDVVYCPRHVICTSE<br>DMLNPNYEDLLIRKSNHNFLVQAGNVQLRVIGHSMQNCVLKLKVDTANPK<br>TPKYKFVRIQPGQTFSVLACYNGSPSGVYQCAMRPNFTIKGSFLNGSCGS<br>VGFNIDYDCVSFCYMHHMELPTGVHAGTDLEGNFYGPFVDRQTAQAAGTD<br>TTITVNVLAWLYAAVINGDRWFLNRFTTTLNDFNLVAMKYNYEPLTQDHV<br>DILGPLSAQTGIAVLDMCASLKELLQNGMNGRTILGSALLEDEFTPFDVV<br>RQCSGVTFQ
**Crystallization condition**	8% (w/v) PEG 4,000<br>0.1 M MES pH 6.7<br>5% (v/v) DMSO	23.5% (w/v) PEG 1,500,<br>0.2 M MIB pH 7.7<br>5% (v/v) DMSO<br>1 mM DTT<br>0.025 mM EDTA pH 7.0
**Soaking condition**		
Chemical composition	8% (w/v) PEG 4,000<br>0.1 M MES pH 6.7<br>5% (v/v) DMSO<br>30% (w/v) PEG 400	23.5% (w/v) PEG 1,500<br>0.2 M MIB pH 7.7<br>5% (v/v) DMSO<br>1 mM DTT<br>0.025 mM EDTA pH 7.0
Maximum fragment concentration	40 mM	100 mM
Soaking time	4h	overnight
**Resolution**	2.2 – 2.8 Å	Average of 1.94 Å
